# Mitochondrial Potassium Channels as Druggable Targets

**DOI:** 10.3390/biom10081200

**Published:** 2020-08-18

**Authors:** Antoni Wrzosek, Bartłomiej Augustynek, Monika Żochowska, Adam Szewczyk

**Affiliations:** Laboratory of Intracellular Ion Channels, Nencki Institute of Experimental Biology, Polish Academy of Sciences, 02-093 Warsaw, Poland; A.Wrzosek@nencki.edu.pl (A.W.); bartlomiej.augustynek@dbmr.unibe.ch (B.A.); M.Zochowska@nencki.edu.pl (M.Ż.)

**Keywords:** mitochondria, potassium channels, ATP, calcium, ROS, potassium channel openers

## Abstract

Mitochondrial potassium channels have been described as important factors in cell pro-life and death phenomena. The activation of mitochondrial potassium channels, such as ATP-regulated or calcium-activated large conductance potassium channels, may have cytoprotective effects in cardiac or neuronal tissue. It has also been shown that inhibition of the mitochondrial Kv1.3 channel may lead to cancer cell death. Hence, in this paper, we examine the concept of the druggability of mitochondrial potassium channels. To what extent are mitochondrial potassium channels an important, novel, and promising drug target in various organs and tissues? The druggability of mitochondrial potassium channels will be discussed within the context of channel molecular identity, the specificity of potassium channel openers and inhibitors, and the unique regulatory properties of mitochondrial potassium channels. Future prospects of the druggability concept of mitochondrial potassium channels will be evaluated in this paper.

## 1. Introduction

The mitochondrial potassium channels field started in the beginning of the 1990s when the first paper describing potassium channel sensitive to ATP and antidiabetic sulphonylurea–glibenclamide (mitoK_ATP_ channel) was described [1]. It was a strong indication that mitochondria may contain potassium channels similar to those present in the plasma membrane. For almost a decade after, it was not clear what the role of mitochondrial potassium channels was. This was because channels would not support, due to membrane potential dissipation, a canonical function of mitochondria i.e., ATP synthesis. The finding of calcium-activated large conductance channel in inner mitochondrial membrane (mitoBK_Ca_) would not help position potassium channel in mitochondrial function [2]. The discovery that activation of the mitochondrial potassium channel by potassium channel openers induces protective mechanisms in cardiac myocytes positioned these proteins as an important player in ischemic preconditioning [3]. This was a starting point for trials to target various mitochondrial potassium channels with drugs to affect cell life.

Potassium channels, which are present in the plasma membrane, are the targets of many substances employed in medicine. This is because K^+^ trafficking through the plasmalemma plays an important role in a variety of processes, including the regulation of heart function, muscle contraction, neurotransmitter release, neuronal excitability, insulin secretion, epithelial electrolyte transport, and cell proliferation [4,5,6]. Hence, plasma membrane potassium channels have been recognized as potential therapeutic drug targets for many years. For example, voltage-regulated potassium channels offer opportunities for the development of new drugs for cancer, autoimmune diseases; and metabolic, neurological, and cardiovascular disorders [4,5,6,7]. Clinically used potassium channel modulators comprise of hypoglycemic sulfonylureas (potassium channel blockers, such as glibenclamide), antiarrhythmic agents (nonselective potassium channel blockers, such as dexsotalol or nifekalant), antianginal and cardioprotective agents (potassium channel openers, such as nicorandil or levosimendan), and anticonvulsants (potassium channel openers, such as retigabine) [8].

The mitochondria, due to their crucial functions within all mammalian cells, are increasingly considered to be targets in drug development [9,10,11,12]. Targeting mitochondrial potassium channels in various cell types is an important aim of future studies in this context. The discovery and characterization of mitochondrial K^+^ channels in the liver, heart, brain, endothelium, or fibroblast cells clarified the mitochondrial K^+^ flux phenomenon [13,14,15,16].

First, the phenomenon of potassium uniport, which has been described previously in mitochondria, is attributed to potassium selective channels [17]. Second, potassium channels in the mitochondrial membrane are similar, due not only to biophysical properties but also pharmacological ones of potassium channels present in the plasma membrane. For example, mitochondrial ATP-regulated potassium (mitoK_ATP_) channels were sensitive not only to ATP but also to antidiabetic sulfonylureas, such as glibenclamide and 5-hydroxydecanoic acid (5-HD), and to potassium channel openers, such as diazoxide [1,18,19,20]. Recently, the molecular identities of mitochondrial (mitoBK_Ca_) and plasma membrane large conductance Ca^2+^-activated potassium (BK_Ca_) channels were determined to have a common genetic origin [21].

To date, potassium ions’ influx into the mitochondria was considered a consequence of high membrane potential (negative in the matrix). Due to K^+^/H^+^ exchanger activity in the inner mitochondrial membrane, no harmful accumulation of K^+^ in the matrix was observed [17]. This kind of description would limit potassium channel action only as part of mitochondrial osmotic controllers. Currently, K^+^ flux is considered to be a regulator of mitochondrial membrane potential and respiration, reactive oxygen species (ROS) synthesis, and mitochondrial plasticity, i.e., morphological changes [6,14,16].

The presence of mitoK_ATP_ channels in the inner mitochondrial membrane suggests that mitochondria may be a target of potassium channel openers known to interact with plasma membrane K_ATP_ channels [22]. Furthermore, linking cytoprotective phenomena with mitochondrial potassium channels (and not with the potassium channel in plasma membrane) gave rise to a new area of research investigating cytoprotective mechanisms via activation of or mitochondrial potassium channels [23]. This finding raised the question of the extent to which mitochondrial potassium channels could be applicable targets for cardiac or neuronal protection in various physiological insults. Interestingly, mitochondrial voltage regulated (mitoKv1.3) channel inhibition was recently described as an early event of cancer cell death [24]. Summarizing, mitochondrial potassium channels’ activation or inhibition by specific drugs could be used in cytoprotective or cell death regulation.

**Figure 1:**
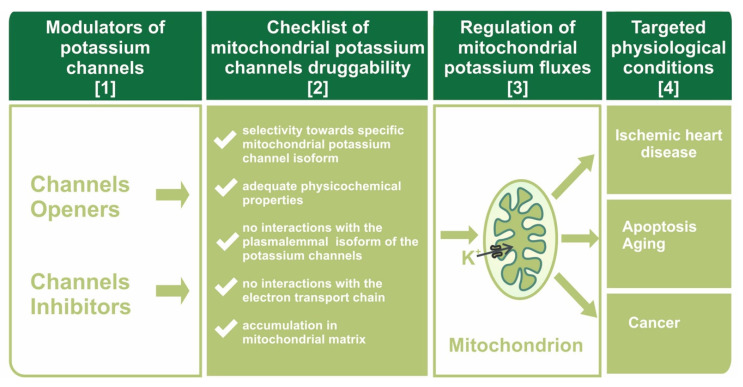
Scheme of the druggability concept towards mitochondrial potassium channels. Modulators of potassium channels (column [1]) must possess unique properties (column [2]) to affect K^+^ flux into the mitochondrial matrix (column [3]) in a specific way, thereby causing beneficial physiological effects (column [4]).

The druggability of mitochondrial potassium channels will be discussed in this paper within the context of channel molecular identity and the specificity of potassium channel openers and inhibitors towards mitochondrial channels (see Figure 1).

The concept of the druggability of mitochondrial potassium channels is also based on drug properties, such as lipophilicity and positive charge, leading to their accumulation in mitochondria. This effect may promote specific drug actions on mitochondrial potassium channels. Moreover, the unique environment for potassium channels in the inner mitochondrial membrane merits critical analysis. Additionally, some pitfalls of using mitochondrial potassium channels as drug targets will be discussed in this paper.

## 2. Three-Dimensional Mitochondrial Potassium Channel Mapping

To consider mitochondrial potassium channels as important targets for drugs, one should first define three parameters. We call this concept three-dimensional mitochondrial potassium channel mapping: “3D mitoK channel mapping”.

The first dimension of “3D mitoK channel mapping” concerns the molecular identity of mitochondrial potassium channels. There are various potassium channels in the inner mitochondrial membrane [16]. Before the era of mitochondrial potassium channels [1], the universal description as “potassium uniport” was used to describe the phenomenon of K^+^ electrogenic influx into the mitochondrial matrix [17]. The current (June 2020) list of identified mitochondrial potassium channels is as follows:

- **mitoK_ATP_ channel:** mitochondrial ATP-regulated potassium channel, the first potassium channel described in the inner mitochondrial membrane [1]. Most likely, these channels might be formed by two proteins: ROMK-type channels (encoded by *KCNJ1* gene) [25,26,27,28] or the recently described CCDC51 protein (encoded by *CCDC51* gene) [29]. Additionally, ABCB8 protein (mitochondrial sulfonylurea receptor) is part of the mitoK_ATP_ channel [29].
- **mitoBK_Ca_ channel** (encoded by *KCNMA1* gene): mitochondrial large conductance calcium-activated potassium channel. This channel is a VEDEC isoform of the BK_Ca_ channel, which is known to be present in the plasma membrane of various cell types [21]
- **mitoIK_Ca_ channel** (encoded by *KCNN4* gene): mitochondrial intermediate conductance calcium-activated potassium channel [30]
- **mitoSK_Ca_ channel** (encoded by *KCNN1, KCNN2,* and *KCNN3* genes): mitochondrial small conductance calcium-activated potassium channel [31]
- **mitoKv1.3 channel** (encoded by *KCNA3* gene): mitochondrial 1.3 voltage-gated potassium channel (the first number denotes subfamily, and the second denotes the order of discovery) [32]
- **mitoKv1.5 channel** (encoded by *KCNA5* gene): mitochondrial 1.5 voltage-gated potassium channel [33]
- **mitoKv7.4 channel** (encoded by *KCNQ4* gene): mitochondrial 7.4 voltage-gated potassium channel [34]
- **mitoTASK3 channel** (encoded by *KCNK9* gene): tandem pore-domain acid-sensitive potassium channel type 3 [35]
- **mitoSLO2** (in *C. elegans* encoded by gene SLO2, in mammals encoded by gene *KCNT2*): mitochondrial sodium-activated potassium channel [36,37]
- **mitoHCN** (encoded by *HCN1, HCN2, HCN3*, and *HCN4* genes): mitochondrial hypopolarization-activated cyclic nucleotide-gated channel [38,39]

The second dimension of “3D mitoK channel mapping” concerns the tissue profile of mitochondrial potassium channels. There is a different set of mitochondrial potassium channels in various tissues [18]. One should not expect that all of the above mentioned channel proteins exist in all types of mitochondria. For example, in cardiomyocyte mitochondria, five different potassium channels were described, but in skin fibroblast mitochondria, only two types of potassium channels were observed [25,40], and in keratinocyte mitochondria, only mitoTASK3 channel was observed [35]. The mitoK_ATP_, mitoBK_Ca_, and mitoKv1.3 channels are the most abundant channels in mammalian mitochondria [15].

The third dimension of “3D mitoK channel mapping” concerns the level (amount) of specific mitochondrial potassium channels in specific tissues. For example, it is believed that the number of mitoK_ATP_ channels is higher in the brain than in cardiac mitochondria [41]. Interestingly, the mitoBK_Ca_ channel in the brain is distributed in various amounts in various brain regions [42].

To summarize, before targeting with drugs specific K^+^ channels in mitochondria, one should consider the following: (1) Type of mitochondrial potassium channel, (2) presence of mitochondrial potassium channels in specific tissues, and (3) the abundance of the mitochondrial potassium channel in targeted tissue versus other tissue.

Additional aspects of this problem concern the presence of mitochondrial channels within various cell compartments. The BK_Ca_ channels, which are highly abundant in the cell membrane of various cells, are present in only the inner mitochondrial membrane (mitoBK_Ca_ channels) but not in plasma membrane of cardiomyocytes [14]. This observation is notably unique for mitochondrial potassium channels and may support that mitochondrial potassium channels and cardiac tissue are druggable targets. The BK_Ca_ channel was also observed in the nucleus membrane [43].

Finally, within the same cell, there are mitochondria both having and not having the potassium channel. It was shown that mitoBK_Ca_ channels are probably not present in all mitochondria within one neuron cell [42]. This uneven distribution of mitoBK_Ca_ channels and the functional consequences of the intracellular heterogeneity of mitochondrial potassium channels still need to be elucidated.

## 3. Plasma Membrane Versus Mitochondrial Potassium Channels: Molecular Identity

Identifying the molecular identity of the mitochondrial potassium channel is a key element for the rational design and application of these proteins as precise drug targets. This step is important because highly similar proteins are both present in the plasma membrane and inner mitochondrial membrane. In recent years, there has been considerable progress in this area, but there are still many open questions [13].

The plasmalemmal ATP-regulated potassium channel (K_ATP_) was first described in cardiomyocytes [44]. This K_ATP_ channel is also present in skeletal muscles, and in pancreas, where it plays a crucial role in the regulation of insulin secretion from β cells. It seems that plasmalemmal K_ATP_ channels have different structures and molecular compositions depending on their localization [45]. The plasmalemmal K_ATP_ channel consists of four Kir6.X pore forming subunits (either Kir6.1 or Kir6.2) and four SUR subunits (SUR1, SUR2, or SUR2B) [46]. K_ATP_ channels are sensitive to changes in ATP concentrations, and by sensing the ATP/ADP ratio in the cytoplasm, these channels possess the unique ability to couple cellular metabolism with plasma membrane potential. As mentioned, K_ATP_ potassium channels of similar electrophysiological and pharmacological properties were also found in the mitochondrial inner membrane: mitoK_ATP_ channels. These channels might be formed by two proteins: ROMK-type channels [26] or CCDC61/ABCB8 protein complex [29].

In fact, the mitoK_ATP_ channel was the first potassium channel ever identified in the mitochondrial inner membrane [1]. This channel was detected in the mitochondria of several tissues, such as the liver, heart, skeletal muscles, and brain [15]. Although the channel’s electrophysiological properties are relatively well described, there is no consensus regarding its molecular topology in the inner mitochondrial membrane. Several groups have investigated this issue, proving that the molecular identity of mitoK_ATP_ is most likely different from its plasmalemmal counterpart. Recent discoveries indicate that the ROMK2 (renal outer medullary potassium channel 2; Kir1.1b) protein is a candidate for the molecular constituent of the mitoK_ATP_ channel [26]. It appears that when overexpressed, the ROMK2 protein tends to localize in the mitochondrial inner membrane. Moreover, shRNA-mediated knockdown of ROMK inhibited ATP-sensitive, diazoxide-activated components of mitochondrial thallium uptake. The ROMK (Kir1.1a) protein was first described in the plasma membrane of renal cells. However, this protein was later found to be widely expressed within different tissues. ROMK proteins are encoded by distinct splice variants of the *Kcnj1* gene. The expression of ROMK in mitochondria and patch-clamp measurements confirmed its functional properties as a mitoK_ATP_ channel [27]. Recently, it was proposed that the CCDC61 and ABCB8 proteins constitute a mitoK_ATP_ channel composed of pore-forming and ATP-binding subunits (mitochondrial sulfonylurea receptor) [29]. Planar lipid bilayer reconstitution of the pore subunit together with the mitochondrial sulfonylurea receptor showed the canonical properties of the mitoK_ATP_ channel. Overexpression of the mitoK_ATP_ channel triggers mitochondrial swelling, whereas genetic ablation of this subunit causes instability in mitochondrial membrane potential and decreases oxidative phosphorylation [29].

The BK_Ca_ (K_Ca_1.1) is ubiquitously expressed within different tissues. The BK_Ca_ channel was first discovered in the plasmalemma of chromaffin cells [47]. However, the activity of the BK_Ca_ channel has also been described in other cellular structures, such as nuclei, ER, or mitochondria (named mitoBK_Ca_ channel) [2]. Expression of the mitoBK_Ca_ channel has been reported in several mammalian cell types, including heart [48], brain [42,49,50,51], skeletal muscle [52], endothelium [53], and fibroblasts [40]. The channel was also found in the mitochondria of certain plants [54] and members of the Protista kingdom [55].

A functional BK_Ca_ channel is composed of four α-subunits. Each α-subunit spanned the membrane seven times. The BK_Ca_ channel represents a unique class of ion channels not only because of its high single channel conductance but also because it can be activated by Ca^2+^ alone, membrane depolarization alone, or by both factors synergistically [56]. This dual regulation allows BK_Ca_ channels to couple intracellular signaling to membrane potential and significantly modulate physiological responses, such as neuronal signaling and muscle contraction [57].

Unlike K_ATP_ channels, all BK_Ca_ channels found in distinct locations within the cell seem to be the products of alternative splicing of a single *Kcnma1* gene [58,59]. Unfortunately, the exact molecular identity of each isoform has not been determined. However, one of the mitochondrial splice variants of the BK_Ca_ channel is believed to have an extended C-terminal domain ending with the amino acid residue VEDEC [60].

It was shown that increased expression of the Kv1.3 potassium (mitoKv1.3) channel in the mitochondria of many types of cancer cells and participation in the process of apoptosis have made it a potential target in cancer therapy [61]. The mitoKv1.3 channel is located on the inner mitochondrial membrane in the same orientation as on the plasma membrane [32]. Similar Kv1.3 channel is also detected in the Golgi apparatus and in the membrane of endoplasmic reticulum. Activation of the Kv1.3 channel located in plasmalemma leads to increased cell proliferation and differentiation. In contrast, the mitoKv1.3 channel plays a key role in activating the apoptotic pathway. CTLL-2 cells overexpressing mitoKv1.3 channels were sensitive to pro-apoptotic factors, such as TNFα, staurosporine, sphingomyelinase, and C6-ceramide. CTLL-2 cells deficient in mitoKv1.3 channels showed no signs of apoptosis, while cells of the same CTLL-2 line with Kv1.3 channels expressed from a mitochondria-targeted vector showed induction of apoptosis in response to TNFα. Induction of apoptosis in mitochondria occurs by blocking the mitoKv1.3 channel through interacting with lysine 128 pro-apoptotic Bax protein, and as a result, the channel pore is impermeable to K^+^ ions. Hyperpolarization of the mitochondrial membrane leads to increased synthesis of reactive oxygen species (ROS) and the release of cytochrome c followed by membrane depolarization and initiation of apoptosis [62].

Molecular definition of mitochondrial potassium channels is important for designing drugs specifically targeting these proteins. This approach probably will help to describe differences between mitochondrial and plasma membrane potassium channels, which is a crucial step in the activity regulation of only mitochondrial potassium channels.

## 4. Unique Regulation of Mitochondrial Potassium Channels: Destination Context?

Mitochondrial potassium channels are regulated by similar factors (as plasma membrane potassium channels) such as ATP, Ca^2+^, ROS, heme, gasotransmitters, or free fatty acids. Additionally, such parameters as membrane potential and/or pH regulate in principle potassium channels in the same way in both the plasma membrane and the mitochondrial inner membrane [15,16,19,63,64,65].

Recently, unique (specific for mitochondria) regulation of mitochondrial potassium channels was reported. The single-channel activity of the mitoBK_Ca_ channel was measured by patch-clamping mitoplasts isolated from the human astrocytoma (glioblastoma) U-87 MG cell line [66]. The channel was activated by Ca^2+^ at micromolar concentrations and by the potassium channel opener NS1619. The channel was inhibited by paxilline and iberiotoxin, which are well characterized inhibitors of BK_Ca_ channels localized in plasmalemma and inner mitochondrial membrane. It was shown that substrates of the respiratory chain, such as NADH, succinate, and glutamate/malate, decrease the activity of the channel at positive voltages. This effect was abolished by rotenone, antimycin, and cyanide, which are inhibitors of the mitochondrial respiratory chain. The putative interaction of the β4 subunit of mitoBK_Ca_ with cytochrome c oxidase (COX) was demonstrated using blue native electrophoresis technique. These results indicated possible structural and functional coupling of the mitoBK_Ca_ channel with the mitochondrial respiratory chain in human astrocytoma U-87 MG cells [66]. Direct regulation of mitoBK_Ca_ channels by mitochondrial respiratory chain redox status may play an important role in the ischemia-reperfusion phenomenon.

The interaction of the mitoBK_Ca_ channel with COX has an additional consequence. It was suggested that mitochondria interact with near-infrared light (wavelengths between 700 and 1400 nm) are absorbed by complexes of the respiratory chain. In the near-infrared region, the 820 nm absorption band belongs mainly to the relatively oxidized Cu_A_ and the 760 nm absorption band to the relatively reduced Cu_B_ chromophore of COX. The absorption of photons (at 760 and 820 nm) by COX is hypothesized to enhance respiratory chain function and increase the synthesis of ATP by mitochondria. The mitoBK_Ca_ channels of the astrocytoma (glioblastoma) U-87 MG cell line were investigated using a patch clamp technique with an illumination system [67]. It was found that the mitoBK_Ca_ channel activity was modulated by illumination by infrared light. Activation of the mitoBK_Ca_ channel (depending on respiratory chain redox state) was observed after illumination using specific light wavelengths: 760 nm or 820 nm. These findings confirmed the functional coupling of the respiratory chain via COX to the mitoBK_Ca_ channel and regulation of its transporting activity by infrared light [67].

Mitochondria are highly dynamic intracellular structures in which, depending on metabolic activity, the inner mitochondrial membrane could be dramatically remodeled. It was shown for the first time that mechanical stimulation of the mitoBK_Ca_ channel resulted in an increased probability of channel opening as was measured by the patch-clamp technique in mitochondria isolated from human astrocytoma U-87 MG cells [68]. These results indicated the possible involvement of the mitoBK_Ca_ channels in mitochondrial activities in which changes in membrane shape and tension play a crucial role, such as fusion/fission and cristae remodeling [68].

These examples illustrate that localization of potassium channels in mitochondrial inner membrane may form a new context of channel regulation. These newly described regulatory mechanisms, as a consequence of mitochondrial localization, may facilitate the design of drugs specifically acting on mitochondrial potassium channels.

## 5. Searching for Specific Drugs Targeting mitoKv1.3 Channels

**Figure 2:**
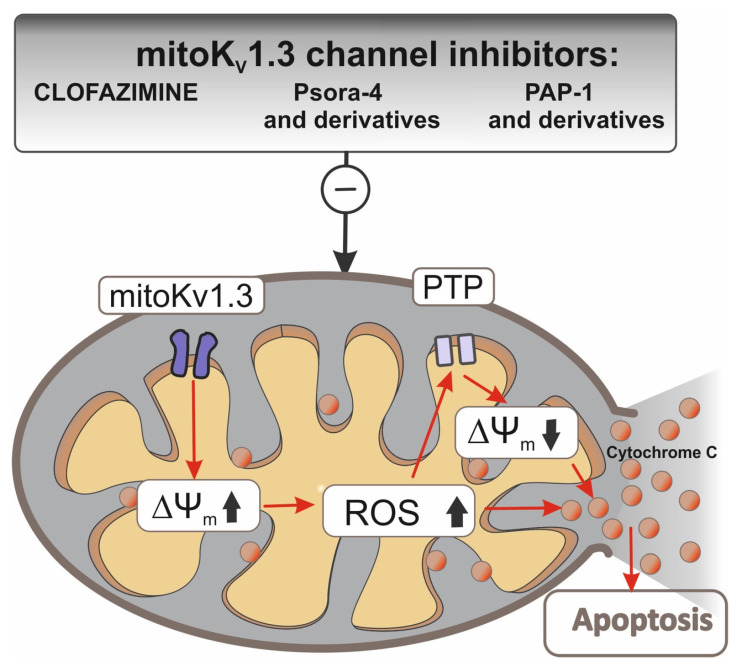
Interactions of Kv1.3 channel inhibitors in mitochondria. Inhibition of the Kv1.3 channel in the inner mitochondrial membrane (IMM) by clofazimin and Psora-4 and PAP-1 and their mitochondrial-directed derivatives (PCTP, PCARBTP, PAPTP) causes hyperpolarization. Hyperpolarization induces an increase in ROS synthesis. If basal ROS production is relatively high as for cancer cells, a further increase in ROS synthesis may lead to a critical level that leads to PTP opening, swelling, and loss of membrane potential, depolarization, and consequent release of cytochrome c from IMM. This chain of events leads to an apoptotic cascade and, as a consequence, to cell death.

Because many cancer cells are deficient in pro-apoptotic proteins, such as Bax or Bak, which causes apoptosis resistance and inhibits the action of chemotherapeutics, it is important to develop a therapy that would cause cancer cells to undergo apoptosis in spite of these deficiencies. The mitochondrial Kv1.3 potassium channel, which is blocked by the Bax protein, has become such a therapeutic target [69]. Inhibitors of this channel have also been shown to be able to activate the internal apoptotic pathway in Bax/Bak deficiency. Three inhibitors were tested: Psora-4, PAP-1, and clofazimine on CTLL-2 cells that do not express Kv1.3 and other potassium channels and on the Kv1.3 transfected line. The three inhibitors blocked the mitochondrial Kv1.3 channel and induced apoptosis only in cells expressing Kv1.3 (Figure 2).

In the case of clofazimine, its pro-apoptotic effect was also tested in vivo in a mouse model of the orthotopic melanoma B16F10 line. A 90% tumor mass reduction after intraperitoneal administration relative to the untreated control was observed with no side effects on healthy tissue [70].

B-cells (B-lymphocytes) from patients suffering from chronic lymphocytic leukemia have been shown to show an increased level of functional mitoKv1.3 channels compared to cells from healthy donors [71]. This finding paved the way for testing the effects of mitoKv1.3 inhibitors, such as Psora-4, PAP-1, and clofazimine, in the treatment of leukemia. These molecules were highly effective in inducing cell death, especially in combination with inhibitors of multidrug resistance (MDR) pumps. Kv1.3 channel-expressing B-cells undergo apoptosis after treatment with Kv1.3 inhibitors, while healthy T cells from the same patient with reduced Kv1.3 channel expression were resistant to the inhibitors used. The selective action of Kv1.3 channel inhibitors depends not only on the level of Kv1.3 channel expression but also on the presence of mild oxidative stress, which sensitizes even a healthy cell to Kv1.3 channel inhibitors, while the pretreatment of B-cell ROS scavengers makes them resistant to the effects of inhibitors [71].

The different functions of the plasmalemma and mitochondrial Kv1.3 channel necessitated the development of selective inhibitors for mitoKv1.3. Two psoralen derivatives (PAP-1) were developed that accumulate in negatively charged mitochondria due to the lipophilic, positively charged triphenylphosphate (TPP^+^) group [72,73]. In PAPTP, the TPP^+^ group is connected by a stable C-C bond. In PCARBTP, it is linked by an ester bond to the PAP-1 core via carbamine. Under physiological conditions, PCARBTP is hydrolyzed to PAPOH. Mitochondriotropic PAP-1 derivatives have been shown to effectively block mitoKv1.3. Many pancreatic ductal adenocarcinoma (PDAC) lines have been shown to overexpress the mitoKv1.3 channel. The MTT test on five PDAC lines showed 90% mortality after using PAPTP and PCARBTP (Table 1). In vivo tests resulted in a 60% reduction in tumor weight and no effect on healthy tissues after using PCARBTP. In addition, it has been shown that the selective apoptotic effect of PAP-1 derivatives on cancer cells, as opposed to effects on healthy cells, is associated not only with increased expression of the mitoKv1.3 channel but also with altered redox status in cancer cells. Increased synthesis of ROS in cancer cells after blocking the mitoKv1.3 channel with a high baseline ROS causes the critical level to be exceeded and the apoptotic path to be initiated [72].

A mitochondrial-targeted Psora-4 derivative called P5TP was obtained in which the distal phenyl ring was replaced by the TPP^+^ group [74]. A new derivative of PAP-1 in which the TPP^+^ group was attached by means of an unstable connection with the carbonate group was named PCTP.

Both derivatives were tested for their effect on viability in Kv1.3-transfected CTLL-2 cells. The use of P5TP did not improve significantly compared to Psora-4 or PAP-1, but PCTP was already effective and selective, and cell viability was dependent on the dose used and was not dependent on the presence of MDR (Multi Drug Resistance) inhibitors. As with previous derivatives, PCTP promoted apoptosis on four PDAC lines in murine melanoma B16-F10 cells by inhibiting the Kv1.3 channel, causing mitochondrial network fragmentation, depolarization, and ROS synthesis, and is a promising drug for in vivo testing [74].

The treatment of brain tumors, particularly glioblastoma (GBM) with Kv 1.3 channel inhibitors, is complex. Although in vitro, experiments on mouse and human GBM lines showed nearly 90% cell mortality after using PAPTP, PCARBTP, and clofazimine; in vivo tests on GBM-implanted mice did not yield any results. The blood-brain barrier (BBB) hinders the achievement of an effective dose in the tumor. It is therefore necessary to find a way to increase the bioavailability of drugs and enable them to pass through the BBB [75].

In summary, mitochondrial Kv1.3 potassium channels appear to be an effective and safe therapeutic target in the treatment of various types of cancer, including those resistant to chemotherapy. Certain difficulties with the bioavailability of mitoKv1.3 inhibitors found in in vivo studies, especially in the case of brain or pancreatic tumors, may be overcome by appropriate structural modifications. These drugs’ efficiency and specificity in relation to cancer cells should be explored in the future.

## 6. Off-Target Action and Drug Repositioning of Potassium Channel Modulators

Since the discovery of potassium channels in eukaryotic cells, a large number of endogenous and synthesized substances have been discovered that modulate potassium channel activity. Due to the similar structure of potassium channels, some of these compounds interact with the channels found in the inner mitochondrial membrane. As mentioned above, a number of plasma membrane modulators, potassium channel openers, and inhibitors have been tested, and some have also been shown to regulate potassium channels located in the inner mitochondrial membrane [18].

Unfortunately, the accumulation of drugs in mitochondria will increase the probability of side effects (off-target effects) on mitochondrial enzymes, especially interactions with the respiratory chain or ATP synthase, which may be harmful due to multiple negative consequences on cellular function [76] (see Table 2). The potassium channel opener diazoxide is still used as the primary treatment to control hypoglycemia in insulinoma [77]. Diazoxide-sensitive K_ATP_ channels were discovered in mitochondria; moreover, it was shown that the mitoK_ATP_ channel is more sensitive to diazoxide than its counterpart in the plasma membrane [20]. It was also observed that diazoxide is responsible for protecting heart cells in the processes of ischemia and reperfusion heart injury [14]. Additionally, diazoxide besides stimulation of the mitoK_ATP_ channel activity has been shown to have protonophoretic properties [78]. It has also been shown that diazoxide is an inhibitor of succinate dehydrogenase (SDH, Complex II) [79,80]. Determining whether the cytoprotective effect of diazoxide is closely related to its effects on mitoK_ATP_ or whether it has a synergistic effect with other targets requires further study. It is possible that diazoxide may exert its cytoprotective effect by inhibiting respiratory chain complex II and producing ROS reactive oxygen species [80,81], or mitoK_ATP_ channels may be involved as an independent factor [82]. Researchers have speculated that targeting nucleotide-requiring enzymes, particularly SDH and cellular ATPases, diazoxide reduces ROS generation and nucleotide degradation, resulting in preservation of tissue ATP levels during ischemia [83].

A similar relationship, as in the case of diazoxide, occurs in the case of the mitoBK_Ca_ channel, for which small molecules NS1619, CGS7184, NS11021, and paxilline, in addition to modulating the activity of channel, affect the activity of a number of proteins associated with the regulation of Ca^2+^ ions and respiratory chain proteins [84,85,86,87].

Notably, CGS7184, BMS191095, and NS1619, which show strong activating properties of the mitoBK_Ca_ potassium channel as measured in the patch-clamp technique administered to the cells and tissue, have the opposite effect [102,103,104]. Thus, despite similar interactions with mitoBK_Ca_, the effect on cell survival is definitely different. In addition to the activation of mitoBK_Ca_, CGS7184 and NS1619 cause an increase of cytosolic calcium ions concentration. The effect is the same for both compounds, whereas the mechanism of Ca^2+^ increase seems to be totally different [85,86]. The potassium channel opener CGS7184 releases Ca^2+^ by interacting with the RyR channel located in the endoplasmic reticulum, while NS1619 releases Ca^2+^ by inhibiting SERCA activity, a Ca^2+^-ATPase, and Ca^2+^ accumulation by endoplasmic reticulum. This difference seems to be important: in the case of CGS7184, the depletion of Ca^2+^ from the ER leads to activation of Ca^2+^-ATPase and ATP hydrolyses, while NS1619 inhibiting SERCA leads to inhibition of ATP depletion by this enzyme [85]. A similar effect targeting cellular ATPases occurs in the presence of the potassium channel opener diazoxide [83]. The mitoBK_Ca_ channel opener NS1619 significantly inhibits the electron transport chain and ATP hydrolysis [105]. An important effect of SERCA inhibition by the potassium channel opener NS1619 is its regulation by pH. At a low pH, SERCA is strongly inhibited by NS1619, and inhibition decreases with increasing pH [85]. Acidification of the cellular environment occurs in ischemia, which changes notably rapidly in the reperfusion and releases an inhibition of SERCA caused by NS1619. SERCA is less sensitive to lower pH and can efficiently hydrolyze ATP during ischemia [105]. Another mitoBK_Ca_ channel opener, NS11021, is highly specific to the mitoBK_Ca_ channel in isolated mitoplast membranes but used in cellular systems accelerate oxygen consumption by cells. It is greatly interesting that besides the activation of mitoBK_Ca_ channels, NS11021 also has strong mitochondrial uncoupling properties [106,107]. NS13558, which is a derivative of NS11021, and to which BK_Ca_ channels are insensitive, has the same property to uncouple the inner mitochondrial membrane and activate the respiratory chain. It is also interesting that NS11021 has a protective effect, despite its uncoupling properties on renal proximal tubular cells from cold storage [108]. It seems that some synergistic effects, in addition to the activation of mitochondrial potassium channels, also play a significant role.

We should also mention that the typical mitoBK_Ca_ channel blocker paxilline has protective effects, independent of channel inhibition, on cellular damage [109]. Paxilline has also been shown to inhibit SERCA at low concentrations, similar to NS1619 [94]. Paxilline has also been shown to reverse the protective effect of NS11021 at low concentrations on cells. This effect, in turn, can be closely related to inhibition of the mitoBK_Ca_ channel [110].

Drug repositioning involves the investigation of existing drugs for new therapeutic applications. Recently, a new set of mitochondrial potassium channels was discovered in skin-derived cells: keratinocytes, dermal fibroblasts, and endothelial cells [25,35,40,53,111]. Naringenin, a plant-derived flavonoid, has been known for many years to have the potential to improve many health problems, such as cardiovascular, metabolic, neurological, and pulmonary disorders; and cancer [112,113]. Recently, the cardioprotective function of naringenin due to activation of the cardiac mitoBK_Ca_ channel was shown [114]. Additionally, with the use of patch-clamp single channel measurements, it was shown in skin fibroblasts that both the mitoK_ATP_ and mitoBK_Ca_ channels were activated by naringenin [111]. These studies suggest that naringenin may function as a potassium channel opener towards mitochondrial potassium channels in skin-derived cells.

## 7. Targeting Drugs into Mitochondria: A Unique Environment for Potassium Channels?

The organelle-specific delivery of drugs is a general problem and modern trend to achieve significant therapeutic effects and minimal off-target effects in molecular pharmacology.

Mitochondria constitute a unique biophysical environment for potassium channels compared to plasma membrane location. These differences may facilitate the search for drugs specific to mitochondrial potassium channels. Mitochondria are the only intracellular organelles with such a high membrane potential (approximately 180–200 mV with a negatively charged matrix). This property promotes accumulation in mitochondrial matrix lipophilic substances being positively charged.

Tetraphenylphosphonium cation (TPP^+^) is a clear example of such a substance. This property was used to measure mitochondrial membrane potential with the use of a TPP^+^ selective electrode. Although several methods can be used to measure the membrane potential in mitochondria, the use of the TPP^+^ selective electrode is still used in many studies with isolated mitochondria due to its sensitivity [115]. Hence, mitochondrial potassium channel openers or inhibitors with properties mentioned above (lipophilic cations or with TPP^+^ moiety) may reflect preference towards mitochondrial potassium channels.

The second unique property of mitochondria concerns matrix pH. Slight alkalization of the mitochondrial matrix (due to respiratory chain activity) will support the accumulation of weak acids in the mitochondrial matrix. Mitochondrial potassium channel openers or inhibitors with weak acid properties may accumulate in the mitochondrial matrix.

These properties constitute a particular attribute of mitochondria as an “antenna” for collecting substances with specific properties and attracting and accumulating them within mitochondria. To what extent these properties may be applied to increase the druggability of mitochondrial potassium channels is a matter of further investigation. Because of the presence of potassium channels in various cellular destinations, it is important to devise new approaches to target drugs into the mitochondria. Targeting mitochondrial potassium channel openers or inhibitors could regulate mitochondrial potassium channels in a more specific and efficient way.

There are various strategies to target drugs into mitochondria [116]. Some of these strategies are based, as previously mentioned, on the use of lipophilic cations, such as TPP^+^, attached to specific molecules (for review see [117]). This kind of mitochondrial targeting was initiated in 1995 with a tri-phenylphosphonium-thiobutyl conjugate as an antioxidant agent. Other lipophilic cations, such as dequalinium and rhodamine 123, were also mitochondria-targeting [118,119,120,121,122]. These cations play the role of “carrier” towards negatively charged mitochondrial matrix.

There are two well-known approaches for mitochondrial drug delivery: direct conjugation of the targeting ligand to drugs and attachment of the targeting ligand to a nanocarrier [116]. Direct drug-targeting ligand conjugation is simple and easy to control, and the drugs can readily reach the mitochondria; however, the conjugation procedure can diminish the biochemical effects within mitochondria. In the case of the nanocarrier system, there is no concern for a loss of therapeutic effect because the physical interaction and solubility issue would be solved, but optimization has remained a challenge due to the use of many different compositions to prepare the nanocarrier. For mitochondrial targeting, some peptides have been prepared and successfully applied based on the cell-penetrating peptide sequence [123]. The mitochondria-targeting peptides (mitochondria-penetrating peptide, mitochondria-targeting sequence, SS peptide, and other peptides) were conjugated with various drugs to improve their therapeutic efficacy. The SS (Szeto-Schiller) peptide antioxidants represent a novel approach with targeted delivery of antioxidants to the inner mitochondrial membrane. The structural motif of these SS peptides centers on alternating aromatic residues and basic amino acids (aromatic-cationic peptides). Mitochondrial targeting sequences (MTSs) can be utilized as vehicles to deliver metalloporphyrin superoxide dismutase (SOD) mimics into the matrix. Recently, thermo responsive drug delivery to mitochondria was described and may represent an interesting and promising technique for cancer therapy [124].

## 8. Concluding Remarks

In this paper, we have described our current understanding of the interactions of numerous drugs with mitochondrial potassium channels. Mitochondria are a unique target for pharmacological intervention due to their high membrane potential and alkaline pH in the matrix. Regulation of the mitochondrial potassium channels by drugs is a complex issue. Increased understanding of the regulation of mitochondrial potassium channels by drugs will not only lead to increased knowledge of mitochondrial channels but may also contribute to the future application of these substances i.e., its druggability. We have also described the secondary effects of the drugs (off-target) in addition to their interaction with their primary target i.e., mitochondrial potassium channels.

The rational pharmacology of mitochondrial potassium channels should be preceded by the molecular identification of these proteins. Identification of the molecular identity of mitochondrial potassium channels will increase insight into the interactions of drugs with mitochondrial potassium channels. This outcome should be possible due to the recent molecular identification of pore-forming and regulatory subunits of mitoK_ATP_ or mitoBK_Ca_ channels. More detailed knowledge would provide more possibilities for the development of therapeutic strategies based on the selective modulation of mitochondrial potassium channels in various tissues. Therapies targeting mitochondrial potassium channels may play an important role in curing a variety of diseases.

In summary, more specific modulators of potassium channels are required for the advanced concept of druggability of mitochondrial potassium channels.

## Figures and Tables

**Table 1:** Compound modulating activity of mitoK_v_1.3 potassium channels.

Chemical IUPAC Name	Abbreviation	Chemical Structure
(3E)-N,5-bis(4-chlorophenyl)-3-isopropylimino-phenazin-2-amine	Clofazimine	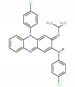
4-(4-phenylbutoxy) furo[3,2-g]chromen-7-one	Psora-4	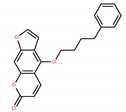
4-(4-phenoxybutoxy)furo[3,2-g]chromen-7-one	PAP-1	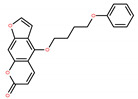
(3-(4-(4-((7-oxo-7H-furo[3,2-g]benzopyran-4-yl)oxy)butoxy)phenyl)propyl)triphenyl phosphonium iodide	PAPTP	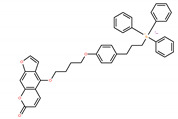
4-[4-(4-hydroxyphenoxy)butoxy]furo[3,2-g]chromen-7-one	PAPOH *	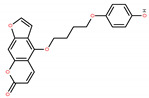
3-[[4-[4-(7-oxofuro[3,2-g]chromen-4-yl)oxybutoxy]phenoxy]carbonylamino]propyl-triphenoxy-phosphonium iodide	PCARBTP	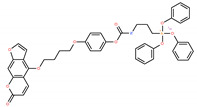
(3-(((4-(4-((7-oxo-7H-furo[3,2-g]chromen-4-yl)oxy)butoxy)phenoxy)carbonyl)oxy) propyl) triphenyl phosphonium iodide	PCTP	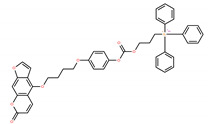
(4-((7-oxo-7H-furo[3,2-g]chromen-4-yl)oxy)butyl) triphenyl phosphonium iodide	P5TP	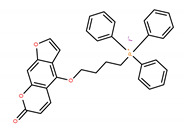

* The compound PAPOH is a product of hydrolysis of PCARBTP and PCTP.

**Table 2:** Off-target action of selected mitochondrial potassium channel modulators.

Mitochondrial Potassium Channels	Potassium Channel Modulators	Off-Target Action
mitoK_ATP_ Channel opener	diazoxide	- SDH inhibitor [79,80] - protonophoric properties [78] - induce translocation of PKC-ξ [88] - increase the expression of p-AKT and p-Foxo1 [89]
Channel blocker	Glibenclamide	- inhibits cardiac cAMP-activated Cl^−^ channels [90] - inhibitors of guinea-pig atrial chloride current [91]
mitoBK_Ca_ Channel openers	NS1619	- SERCA inhibition [85] - inhibition L-type calcium channels [92] - stimulate Ca^2+^-gated chloride currents [93]
	CGS7184	- RyR channel inhibition by CGS7184 [86,87]
Channel blocker	paxilline	-modulation of the ATP-dependent Ca^2+^-ATPase at the phosphoenzyme level [94]
mitoK_V_1.3 Channel blocker	Clofazimine	- inhibitor of acid sphingomyelinase [95]
mitoK_V_ 7.4 Channel opener	Retigabine	- interaction with GABAergic and glutamatergic neurotransmission [96]
mitoTASK3 Channel openers	Halothane	-inhibition of synthesis of 5-hydroxytryptamine [97]
	Terbinafine	- CYP2D6 inhibition [98]
Channel blocker	Lidocaine	-interaction with Ca-ATPase in cardiac sarcoplasmic reticulum [99]
mitoHCN Channel blocker	ZD7288	- reduce T-type calcium channel currents [100] - inhibitor Na^+^ current [101]

## References

[1] Inoue I., Nagase H., Kishi K., Higuti T. (1991). ATP-sensitive K^+^ channel in the mitochondrial inner membrane. Nature.

[2] Siemen D., Loupatatzis C., Borecky J., Gulbins E., Lang F. (1999). Ca^2+^-activated K channel of the BK-type in the inner mitochondrial membrane of a human glioma cell line. Biochem. Biophys. Res. Commun..

[3] Liu Y., Sato T., O’Rourke B., Marban E. (1998). Mitochondrial ATP-dependent potassium channels: Novel effectors of cardioprotection?. Circulation.

[4] Crotti L., Odening K.E., Sanguinetti M.C. (2020). Heritable arrhythmias associated with abnormal function of cardiac potassium channels. Cardiovasc. Res..

[5] D’Adamo M.C., Liantonio A., Rolland J.F., Pessia M., Imbrici P. (2020). Kv1.1 Channelopathies: Pathophysiological Mechanisms and Therapeutic Approaches. Int. J. Mol. Sci..

[6] Trombetta-Lima M., Krabbendam I.E., Dolga A.M. (2020). Calcium-activated potassium channels: Implications for aging and age-related neurodegeneration. Int. J. Biochem. Cell Biol..

[7] Zhang L., Zheng Y., Xie J., Shi L. (2020). Potassium channels and their emerging role in parkinson’s disease. Brain Res. Bull..

[8] Walsh K.B. (2020). Screening Technologies for Inward Rectifier Potassium Channels: Discovery of New Blockers and Activators. SLAS Discov..

[9] Moos W.H., Dykens J.A. (2015). Mitochondrial drugs come of age. Drug Dev. Res..

[10] Olszewska A., Szewczyk A. (2013). Mitochondria as a pharmacological target: Magnum overview. IUBMB Life.

[11] Stoker M.L., Newport E., Hulit J.C., West A.P., Morten K.J. (2019). Impact of pharmacological agents on mitochondrial function: A growing opportunity?. Biochem. Soc. Trans..

[12] Szewczyk A., Wojtczak L. (2002). Mitochondria as a Pharmacological Target. Pharmacol. Rev..

[13] Laskowski M., Augustynek B., Kulawiak B., Koprowski P., Bednarczyk P., Jarmuszkiewicz W., Szewczyk A. (2016). What do we not know about mitochondrial potassium channels?. Biochim. Biophys. Acta (BBA)-Bioenerg..

[14] O’Rourke B. (2004). Evidence for mitochondrial K^+^ channels and their role in cardioprotection. Circ. Res..

[15] Szabo I., Zoratti M. (2014). Mitochondrial channels: Ion fluxes and more. Physiol. Rev..

[16] Szewczyk A., Jarmuszkiewicz W., Kunz W.S. (2009). Mitochondrial potassium channels. IUBMB Life.

[17] Garlid K.D., Paucek P. (2003). Mitochondrial potassium transport: The K^+^ cycle. Biochim. Biophys. Acta.

[18] Augustynek B., Kunz W.S., Szewczyk A. (2017). Guide to the Pharmacology of Mitochondrial Potassium Channels. Handb. Exp. Pharmacol..

[19] Leanza L., Checchetto V., Biasutto L., Rossa A., Costa R., Bachmann M., Zoratti M., Szabo I. (2019). Pharmacological modulation of mitochondrial ion channels. Br. J. Pharmacol..

[20] Paucek P., Mironova G., Mahdi F., Beavis A.D., Woldegiorgis G., Garlid K.D. (1992). Reconstitution and partial purification of the glibenclamide-sensitive, ATP-dependent K^+^ channel from rat liver and beef heart mitochondria. J. Biol. Chem..

[21] Singh H., Lu R., Bopassa J.C., Meredith A.L., Stefani E., Toro L. (2013). mitoBK_Ca_ is encoded by the *Kcnma1* gene, and a splicing sequence defines its mitochondrial location. Proc. Natl. Acad. Sci. USA.

[22] Szewczyk A., Marban E. (1999). Mitochondria: A new target for K channel openers?. Trends Pharmacol. Sci..

[23] Hausenloy D.J., Schulz R., Girao H., Kwak B.R., De Stefani D., Rizzuto R., Bernardi P., Di Lisa F. (2020). Mitochondrial ion channels as targets for cardioprotection. J. Cell Mol. Med..

[24] Peruzzo R., Mattarei A., Romio M., Paradisi C., Zoratti M., Szabo I., Leanza L. (2017). Regulation of Proliferation by a Mitochondrial Potassium Channel in Pancreatic Ductal Adenocarcinoma Cells. Front. Oncol..

[25] Bednarczyk P., Kicinska A., Laskowski M., Kulawiak B., Kampa R., Walewska A., Krajewska M., Jarmuszkiewicz W., Szewczyk A. (2018). Evidence for a mitochondrial ATP-regulated potassium channel in human dermal fibroblasts. Biochim. Biophys. Acta Bioenerg..

[26] Foster D.B., Ho A.S., Rucker J., Garlid A.O., Chen L., Sidor A., Garlid K.D., O’Rourke B. (2012). Mitochondrial ROMK channel is a molecular component of mitoK(ATP). Circ. Res..

[27] Laskowski M., Augustynek B., Bednarczyk P., Zochowska M., Kalisz J., O’Rourke B., Szewczyk A., Kulawiak B. (2019). Single-Channel Properties of the ROMK-Pore-Forming Subunit of the Mitochondrial ATP-Sensitive Potassium Channel. Int. J. Mol. Sci..

[28] Papanicolaou K.N., Ashok D., Liu T., Bauer T.M., Sun J., Li Z., da Costa E., D’Orleans C.C., Nathan S., Lefer D.J. (2020). Global knockout of ROMK potassium channel worsens cardiac ischemia-reperfusion injury but cardiomyocyte-specific knockout does not: Implications for the identity of mitoK_ATP_. J. Mol. Cell Cardiol..

[29] Paggio A., Checchetto V., Campo A., Menabò R., Di Marco G., Di Lisa F., Szabo I., Rizzuto R., De Stefani D. (2019). Identification of an ATP-sensitive potassium channel in mitochondria. Nature.

[30] De Marchi U., Sassi N., Fioretti B., Catacuzzeno L., Cereghetti G.M., Szabo I., Zoratti M. (2009). Intermediate conductance Ca^2+^-activated potassium channel (K_Ca_3.1) in the inner mitochondrial membrane of human colon cancer cells. Cell Calcium..

[31] Dolga A.M., Netter M.F., Perocchi F., Doti N., Meissner L., Tobaben S., Grohm J., Zischka H., Plesnila N., Decher N. (2013). Mitochondrial small conductance SK2 channels prevent glutamate-induced oxytosis and mitochondrial dysfunction. J. Biol. Chem..

[32] Szabo I., Bock J., Jekle A., Soddemann M., Adams C., Lang F., Zoratti M., Gulbins E. (2005). A novel potassium channel in lymphocyte mitochondria. J. Biol. Chem..

[33] Leanza L., Zoratti M., Gulbins E., Szabo I. (2012). Induction of apoptosis in macrophages via Kv1.3 and Kv1.5 potassium channels. Curr. Med. Chem..

[34] Testai L., Barrese V., Soldovieri M.V., Ambrosino P., Martelli A., Vinciguerra I., Miceli F., Greenwood I.A., Curtis M.J., Breschi M.C. (2016). Expression and function of Kv7.4 channels in rat cardiac mitochondria: Possible targets for cardioprotection. Cardiovasc. Res..

[35] Toczylowska-Maminska R., Olszewska A., Laskowski M., Bednarczyk P., Skowronek K., Szewczyk A. (2014). Potassium channel in the mitochondria of human keratinocytes. J. Invest. Dermatol..

[36] Wojtovich A.P., Smith C.O., Urciuoli W.R., Wang Y.T., Xia X.M., Brookes P.S., Nehrke K. (2016). Cardiac Slo2.1 Is Required for Volatile Anesthetic Stimulation of K+ Transport and Anesthetic Preconditioning. Anesthesiology.

[37] Wojtovich A.P., Sherman T.A., Nadtochiy S.M., Urciuoli W.R., Brookes P.S., Nehrke K. (2011). SLO-2 is cytoprotective and contributes to mitochondrial potassium transport. PLoS ONE.

[38] León-Aparicio D., Salvador C., Aparicio-Trejo O.E., Briones-Herrera A., Pedraza-Chaverri J., Vaca L., Sampieri A., Padilla-Flores T., López-González Z., León-Contreras J.C. (2019). Novel Potassium Channels in Kidney Mitochondria: The Hyperpolarization-Activated and Cyclic Nucleotide-Gated HCN Channels. Int. J. Mol. Sci..

[39] Padilla-Flores T., López-González Z., Vaca L., Aparicio-Trejo O.E., Briones-Herrera A., Riveros-Rosas H., Pedraza-Chaverri J., León-Aparicio D., Salvador C., Sampieri A. (2020). “Funny” channels in cardiac mitochondria modulate membrane potential and oxygen consumption. Biochem. Biophys. Res. Commun..

[40] Kicinska A., Augustynek B., Kulawiak B., Jarmuszkiewicz W., Szewczyk A., Bednarczyk P. (2016). A large-conductance calcium-regulated K^+^ channel in human dermal fibroblast mitochondria. Biochem. J..

[41] Garlid K.D., Halestrap A.P. (2012). The mitochondrial K_ATP_ channel—Fact or fiction?. J. Mol. Cell Cardiol..

[42] Piwonska M., Wilczek E., Szewczyk A., Wilczynski G.M. (2008). Differential distribution of Ca^2+^-activated potassium channel beta4 subunit in rat brain: Immunolocalization in neuronal mitochondria. Neuroscience.

[43] Li B., Jie W., Huang L., Wei P., Li S., Luo Z., Friedman A.K., Meredith A.L., Han M.-H., Zhu X.-H. (2014). Nuclear BK channels regulate gene expression via the control of nuclear calcium signaling. Nat. Neurosci..

[44] Noma A. (1983). ATP-regulated K^+^ channels in cardiac muscle. Nature.

[45] Tinker A., Aziz Q., Li Y., Specterman M. (2018). ATP-Sensitive Potassium Channels and Their Physiological and Pathophysiological Roles. Compr. Physiol..

[46] Inagaki N., Gonoi T., Clement J.P.T., Namba N., Inazawa J., Gonzalez G., Aguilar-Bryan L., Seino S., Bryan J. (1995). Reconstitution of IK_ATP_: AN inward rectifier subunit plus the sulfonylurea receptor. Science.

[47] Marty A. (1981). Ca-dependent K channels with large unitary conductance in chromaffin cell membranes. Nature.

[48] Xu W., Liu Y., Wang S., McDonald T., Van Eyk J.E., Sidor A., O’Rourke B. (2002). Cytoprotective role of Ca^2+^- activated K^+^ channels in the cardiac inner mitochondrial membrane. Science.

[49] Kulawiak B., Bednarczyk P. (2005). Reconstitution of brain mitochondria inner membrane into planar lipid bilayer. Acta Neurobiol. Exp..

[50] Singh H., Li M., Hall L., Chen S., Sukur S., Lu R., Caputo A., Meredith A.L., Stefani E., Toro L. (2016). MaxiK channel interactome reveals its interaction with GABA transporter 3 and heat shock protein 60 in the mammalian brain. Neuroscience.

[51] Skalska J., Bednarczyk P., Piwonska M., Kulawiak B., Wilczynski G., Dolowy K., Kudin A.P., Kunz W.S., Szewczyk A. (2009). Calcium ions regulate K^+^ uptake into brain mitochondria: The evidence for a novel potassium channel. Int. J. Mol. Sci..

[52] Skalska J., Piwonska M., Wyroba E., Surmacz L., Wieczorek R., Koszela-Piotrowska I., Zielinska J., Bednarczyk P., Dolowy K., Wilczynski G.M. (2008). A novel potassium channel in skeletal muscle mitochondria. Biochim. Biophys. Acta.

[53] Bednarczyk P., Koziel A., Jarmuszkiewicz W., Szewczyk A. (2013). Large-conductance Ca^2+^-activated potassium channel in mitochondria of endothelial EA.hy926 cells. Am. J. Physiol. Heart Circ. Physiol..

[54] Koszela-Piotrowska I., Matkovic K., Szewczyk A., Jarmuszkiewicz W. (2009). A large-conductance calcium-activated potassium channel in potato (Solanum tuberosum) tuber mitochondria. Biochem. J..

[55] Laskowski M., Kicinska A., Szewczyk A., Jarmuszkiewicz W. (2015). Mitochondrial large-conductance potassium channel from Dictyostelium discoideum. Int. J. Biochem. Cell Biol..

[56] Magleby K.L. (2003). Gating mechanism of BK (Slo1) channels: So near, yet so far. J. Gen. Physiol..

[57] Nardi A., Olesen S.P. (2008). BK channel modulators: A comprehensive overview. Curr. Med. Chem..

[58] Latorre R., Brauchi S. (2006). Large conductance Ca^2+^-activated K^+^ (BK) channel: Activation by Ca^2+^ and voltage. Biol. Res..

[59] Sakai Y., Harvey M., Sokolowski B. (2011). Identification and quantification of full-length BK channel variants in the developing mouse cochlea. J. Neurosci. Res..

[60] Ahmad T., Mukherjee S., Pattnaik B., Kumar M., Singh S., Kumar M., Rehman R., Tiwari B.K., Jha K.A., Barhanpurkar A.P. (2014). Miro1 regulates intercellular mitochondrial transport & enhances mesenchymal stem cell rescue efficacy. EMBO J..

[61] Gulbins E., Sassi N., Grassme H., Zoratti M., Szabo I. (2010). Role of Kv1.3 mitochondrial potassium channel in apoptotic signalling in lymphocytes. Biochim. Biophys. Acta.

[62] Szabo I., Bock J., Grassme H., Soddemann M., Wilker B., Lang F., Zoratti M., Gulbins E. (2008). Mitochondrial potassium channel Kv1.3 mediates Bax-induced apoptosis in lymphocytes. Proc. Natl. Acad. Sci. USA.

[63] Rotko D., Bednarczyk P., Koprowski P., Kunz W.S., Szewczyk A., Kulawiak B. (2020). Heme is required for carbon monoxide activation of mitochondrial BKCa channel. Eur. J. Pharmacol..

[64] Rotko D., Kunz W.S., Szewczyk A., Kulawiak B. (2020). Signaling pathways targeting mitochondrial potassium channels. Int. J. Biochem. Cell Biol..

[65] Walewska A., Szewczyk A., Koprowski P. (2018). Gas Signaling Molecules and Mitochondrial Potassium Channels. Int. J. Mol. Sci..

[66] Bednarczyk P., Wieckowski M.R., Broszkiewicz M., Skowronek K., Siemen D., Szewczyk A. (2013). Putative Structural and Functional Coupling of the Mitochondrial BK Channel to the Respiratory Chain. PLoS ONE.

[67] Szewczyk A., Bednarczyk P. (2018). Modulation of the Mitochondrial Potassium Channel Activity by Infrared Light. Biophys. J..

[68] Walewska A., Kulawiak B., Szewczyk A., Koprowski P. (2018). Mechanosensitivity of mitochondrial large-conductance calcium-activated potassium channels. Biochim. Biophys. Acta Bioenerg..

[69] Checchetto V., Prosdocimi E., Leanza L. (2019). Mitochondrial Kv1.3: A New Target in Cancer Biology?. Cell Physiol. Biochem..

[70] Leanza L., Henry B., Sassi N., Zoratti M., Chandy K.G., Gulbins E., Szabo I. (2012). Inhibitors of mitochondrial Kv1.3 channels induce Bax/Bak-independent death of cancer cells. EMBO Mol. Med..

[71] Leanza L., Trentin L., Becker K.A., Frezzato F., Zoratti M., Semenzato G., Gulbins E., Szabo I. (2013). Clofazimine, Psora-4 and PAP-1, inhibitors of the potassium channel Kv1.3, as a new and selective therapeutic strategy in chronic lymphocytic leukemia. Leukemia.

[72] Leanza L., Romio M., Becker K.A., Azzolini M., Trentin L., Manago A., Venturini E., Zaccagnino A., Mattarei A., Carraretto L. (2017). Direct Pharmacological Targeting of a Mitochondrial Ion Channel Selectively Kills Tumor Cells In Vivo. Cancer Cell.

[73] Teisseyre A., Palko-Labuz A., Sroda-Pomianek K., Michalak K. (2019). Voltage-Gated Potassium Channel Kv1.3 as a Target in Therapy of Cancer. Front. Oncol..

[74] Mattarei A., Romio M., Manago A., Zoratti M., Paradisi C., Szabo I., Leanza L., Biasutto L. (2018). Novel Mitochondria-Targeted Furocoumarin Derivatives as Possible Anti-Cancer Agents. Front. Oncol..

[75] Venturini E., Leanza L., Azzolini M., Kadow S., Mattarei A., Weller M., Tabatabai G., Edwards M.J., Zoratti M., Paradisi C. (2017). Targeting the Potassium Channel Kv1.3 Kills Glioblastoma Cells. Neurosignals.

[76] Szewczyk A., Kajma A., Malinska D., Wrzosek A., Bednarczyk P., Zablocka B., Dolowy K. (2010). Pharmacology of mitochondrial potassium channels: Dark side of the field. FEBS Lett..

[77] Gilliaux Q., Bertrand C., Hanon F., Donckier J.E. (2020). Preoperative treatment of benign insulinoma: Diazoxide or somatostatin analogues?. Acta Chir. Belg..

[78] Kowaltowski A.J., Seetharaman S., Paucek P., Garlid K.D. (2001). Bioenergetic consequences of opening the ATP-sensitive K^+^ channel of heart mitochondria. Am. J. Physiol. Heart Circ. Physiol..

[79] Akopova O., Kolchinskaya L., Nosar V., Mankovska I., Sagach V. (2020). Diazoxide affects mitochondrial bioenergetics by the opening of mKATP channel on submicromolar scale. BMC Mol. Cell Biol..

[80] Drose S., Brandt U., Hanley P.J. (2006). K^+^-independent actions of diazoxide question the role of inner membrane K_ATP_ channels in mitochondrial cytoprotective signaling. J. Biol. Chem..

[81] Hanley P.J., Mickel M., Loffler M., Brandt U., Daut J. (2002). K_ATP_ channel-independent targets of diazoxide and 5-hydroxydecanoate in the heart. J. Physiol..

[82] Dröse S., Bleier L., Brandt U. (2011). A Common Mechanism Links Differently Acting Complex II Inhibitors to Cardioprotection: Modulation of Mitochondrial Reactive Oxygen Species Production. Mol. Pharmacol..

[83] Dzeja P.P., Bast P., Ozcan C., Valverde A., Holmuhamedov E.L., Van Wylen D.G., Terzic A. (2003). Targeting nucleotide-requiring enzymes: Implications for diazoxide-induced cardioprotection. Am. J. Physiol. Heart Circ. Physiol..

[84] Debska G., Kicinska A., Dobrucki J., Dworakowska B., Nurowska E., Skalska J., Dolowy K., Szewczyk A. (2003). Large-conductance K^+^ channel openers NS1619 and NS004 as inhibitors of mitochondrial function in glioma cells. Biochem. Pharmacol..

[85] Wrzosek A. (2014). The potassium channel opener NS1619 modulates calcium homeostasis in muscle cells by inhibiting SERCA. Cell Calcium..

[86] Wrzosek A., Tomaskova Z., Ondrias K., Łukasiak A., Szewczyk A. (2012). The potassium channel opener CGS7184 activates Ca^2+^ release from the endoplasmic reticulum. Eur. J. Pharmacol..

[87] Wrzosek A., Tomaskova Z., Ondrias K., Łukasiak A., Szewczyk A. (2012). CGS7184 a potassium channel opener modulates activity of mitochondria and Ca^2+^ homeostasis. Biochim. Biophys. Acta (BBA)– Bioenerg..

[88] Kim M.Y., Kim M.J., Yoon I.S., Ahn J.H., Lee S.H., Baik E.J., Moon C.H., Jung Y.S. (2006). Diazoxide acts more as a PKC-Epsilon activator, and indirectly activates the mitochondrial K(ATP) channel conferring cardioprotection against hypoxic injury. Br. J. Pharmacol..

[89] Duan P., Wang J., Li Y., Wei S., Su F., Zhang S., Duan Y., Wang L., Zhu Q. (2018). Opening of mitoKATP improves cardiac function and inhibits apoptosis via the AKT-Foxo1 signaling pathway in diabetic cardiomyopathy. Int. J. Mol. Med..

[90] Tominaga M., Horie M., Sasayama S., Okada Y. (1995). Glibenclamide, an ATP-sensitive K^+^ channel blocker, inhibits cardiac cAMP-activated Cl- conductance. Circ. Res..

[91] Sakaguchi M., Matsuura H., Ehara T. (1997). Swelling-induced Cl- current in guinea-pig atrial myocytes: Inhibition by glibenclamide. J. Physiol..

[92] Park W.S., Kang S.H., Son Y.K., Kim N., Ko J.-H., Kim H.K., Ko E.A., Kim C.D., Han J. (2007). The mitochondrial Ca^2+^-activated K^+^ channel activator, NS 1619 inhibits L-type Ca^2+^ channels in rat ventricular myocytes. Biochem. Biophys. Res. Commun..

[93] Saleh S.N., Angermann J.E., Sones W.R., Leblanc N., Greenwood I.A. (2007). Stimulation of Ca^2+^-gated Cl^-^ currents by the calcium-dependent K^+^ channel modulators NS1619 [1,3-dihydro-1-[2-hydroxy-5-(trifluoromethyl)phenyl]-5-(trifluoromethyl)-2 H-benzimidazol-2-one] and isopimaric acid. J. Pharmacol. Exp. Ther..

[94] Bilmen J.G., Wootton L.L., Michelangeli F. (2002). The mechanism of inhibition of the sarco/endoplasmic reticulum Ca^2+^ ATPase by paxilline. Arch. Biochem. Biophys..

[95] Kornhuber J., Muehlbacher M., Trapp S., Pechmann S., Friedl A., Reichel M., Mühle C., Terfloth L., Groemer T.W., Spitzer G.M. (2011). Identification of novel functional inhibitors of acid sphingomyelinase. PloS ONE.

[96] Rundfeldt C., Netzer R. (2000). Investigations into the mechanism of action of the new anticonvulsant retigabine—Interaction with GABAergic and glutamatergic neurotransmission and with voltage gated ion channels. Arzneimittelforschung.

[97] De Crescenzo V., Dubuis E., Constantin S., Rebocho M., Girardin C., Bonnet P., Vandier C. (2001). Halothane differentially decreases 5-hydroxytryptamine-induced contractions in normal and chronic hypoxic rat pulmonary arteries. Acta Physiol. Scand..

[98] Madani S., Barilla D., Cramer J., Wang Y., Paul C. (2002). Effect of terbinafine on the pharmacokinetics and pharmacodynamics of desipramine in healthy volunteers identified as cytochrome P450 2D6 (CYP2D6) extensive metabolizers. J. Clin. Pharmacol..

[99] Karon B.S., Geddis L.M., Kutchai H., Thomas D.D. (1995). Anesthetics alter the physical and functional properties of the Ca-ATPase in cardiac sarcoplasmic reticulum. Biophys. J..

[100] Sánchez-Alonso J.L., Halliwell J.V., Colino A. (2008). ZD 7288 inhibits T-type calcium current in rat hippocampal pyramidal cells. Neurosci. Lett..

[101] Wu X., Liao L., Liu X., Luo F., Yang T., Li C. (2012). Is ZD7288 a selective blocker of hyperpolarization-activated cyclic nucleotide-gated channel currents?. Channels.

[102] Augustynek B., Koprowski P., Rotko D., Kunz W.S., Szewczyk A., Kulawiak B. (2018). Mitochondrial BK Channel Openers CGS7181 and CGS7184 Exhibit Cytotoxic Properties. Int. J. Mol. Sci..

[103] Chmielewska L., Malinska D. (2011). Cytoprotective action of the potassium channel opener NS1619 under conditions of disrupted calcium homeostasis. Pharmacol. Rep..

[104] Malinska D., Kulawiak B., Wrzosek A., Kunz W.S., Szewczyk A. (2010). The cytoprotective action of the potassium channel opener BMS-191095 in C2C12 myoblasts is related to the modulation of calcium homeostasis. Cell Physiol. Biochem..

[105] Łukasiak A., Skup A., Chlopicki S., Łomnicka M., Kaczara P., Proniewski B., Szewczyk A., Wrzosek A. (2016). SERCA, complex I of the respiratory chain and ATP-synthase inhibition are involved in pleiotropic effects of NS1619 on endothelial cells. Eur. J. Pharmacol..

[106] Bentzen B.H., Andersen R.W., Olesen S.P., Grunnet M., Nardi A. (2010). Synthesis and characterisation of NS13558: A new important tool for addressing KCa1.1 channel function ex vivo. Naunyn Schmiedebergs Arch. Pharmacol..

[107] Bentzen B.H., Nardi A., Calloe K., Madsen L.S., Olesen S.r.-P., Grunnet M. (2007). The Small Molecule NS11021 Is a Potent and Specific Activator of Ca^2+^-Activated Big-Conductance K^+^ Channels. Mol. Pharmacol..

[108] Shrum S., Rusch N.J., MacMillan-Crow L.A. (2019). Specific BK Channel Activator NS11021 Protects Rat Renal Proximal Tubular Cells from Cold Storage-Induced Mitochondrial Injury In Vitro. Biomolecules.

[109] Kulawiak B., Szewczyk A. (2012). Glutamate-induced cell death in HT22 mouse hippocampal cells is attenuated by paxilline, a BK channel inhibitor. Mitochondrion.

[110] Bentzen B.H., Osadchii O., Jespersen T., Hansen R.S., Olesen S.P., Grunnet M. (2009). Activation of big conductance Ca^2+^-activated K^+^ channels (BK) protects the heart against ischemia-reperfusion injury. Pflug. Arch..

[111] Kampa R.P., Kicinska A., Jarmuszkiewicz W., Pasikowska-Piwko M., Dolegowska B., Debowska R., Szewczyk A., Bednarczyk P. (2019). Naringenin as an opener of mitochondrial potassium channels in dermal fibroblasts. Exp. Dermatol..

[112] Fuster M.G., Carissimi G., Montalban M.G., Villora G. (2020). Improving Anticancer Therapy with Naringenin-Loaded Silk Fibroin Nanoparticles. Nanomaterials.

[113] Rivoira M.A., Rodriguez V., Talamoni G., de Talamoni N.T. (2020). New perspectives in the pharmacological potential of naringin in medicine. Curr. Med. Chem..

[114] Testai L., Da Pozzo E., Piano I., Pistelli L., Gargini C., Breschi M.C., Braca A., Martini C., Martelli A., Calderone V. (2017). The Citrus Flavanone Naringenin Produces Cardioprotective Effects in Hearts from 1 Year Old Rat, through Activation of mitoBK Channels. Front. Pharmacol..

[115] Teodoro J.S., Palmeira C.M., Rolo A.P. (2018). Mitochondrial Membrane Potential (DeltaPsi) Fluctuations Associated with the Metabolic States of Mitochondria. Methods Mol. Biol..

[116] Heller A., Brockhoff G., Goepferich A. (2012). Targeting drugs to mitochondria. Eur. J. Pharm. Biopharm..

[117] Battogtokh G., Cho Y.Y., Lee J.Y., Lee H.S., Kang H.C. (2018). Mitochondrial-Targeting Anticancer Agent Conjugates and Nanocarrier Systems for Cancer Treatment. Front. Pharmacol..

[118] Christman J.E., Miller D.S., Coward P., Smith L.H., Teng N.N. (1990). Study of the selective cytotoxic properties of cationic, lipophilic mitochondrial-specific compounds in gynecologic malignancies. Gynecol. Oncol..

[119] Dairkee S.H., Hackett A.J. (1991). Differential retention of rhodamine 123 by breast carcinoma and normal human mammary tissue. Breast Cancer Res. Treat..

[120] Ernster L., Schatz G. (1981). Mitochondria: A historical review. J. Cell Biol..

[121] Lampidis T.J., Munck J.N., Krishan A., Tapiero H. (1985). Reversal of resistance to rhodamine 123 in adriamycin-resistant Friend leukemia cells. Cancer Res..

[122] Weiss M.J., Wong J.R., Ha C.S., Bleday R., Salem R.R., Steele G.D., Chen L.B. (1987). Dequalinium, a topical antimicrobial agent, displays anticarcinoma activity based on selective mitochondrial accumulation. Proc. Natl. Acad. Sci. USA.

[123] Szeto H.H. (2006). Cell-permeable, mitochondrial-targeted, peptide antioxidants. AAPS J..

[124] Ruan L., Zhou M., Chen J., Huang H., Zhang J., Sun H., Chai Z., Hu Y. (2019). Thermoresponsive drug delivery to mitochondria in vivo. Chem. Commun..

